# Use of fall risk increasing drugs in residents of retirement villages: a pilot study of long term care and retirement home residents in Ontario, Canada

**DOI:** 10.1186/s13104-015-1557-2

**Published:** 2015-10-14

**Authors:** Carlos Rojas-Fernandez, Farzan Dadfar, Andrea Wong, Susan G. Brown

**Affiliations:** Schlegel Research Chair in Geriatric Pharmacotherapy, Schlegel-UW Research Institute for Ageing, School of Pharmacy, University of Waterloo, 10 Victoria St S, Room 7004, Kitchener, ON N2G 1C5 Canada; University of Waterloo School of Pharmacy, Kitchener, ON Canada; Schlegel-University of Waterloo Research Institute for Aging, 325 Max Becker Drive, Suite 202, Kitchener, ON N2E 4H5 Canada; http://www.the-ria.ca

**Keywords:** Geriatrics, Falls, Psychotropics, Antihypertensives, Fall-risk increasing drugs, Narcotics, Long-term care, Assisted living

## Abstract

**Background:**

Falls continue to be a problem for older people in long-term care (LTC) and retirement home (RH) settings and are associated with significant morbidity and health care use. Fall-risk increasing drugs (FRIDs) are known to increase fall risk and represent modifiable risk factors. There are limited data regarding the use of FRIDs in contemporary LTC and RH settings, and it has not been well documented to what extent medication regimens are reviewed and modified for those who have sustained falls. The objective of this study is to characterize medication related fall risk factors in LTC and RH residents and on-going use of medications known to increase fall risk.

**Methods:**

Retrospective chart review of residents aged >65 who sustained one or more falls living in LTC or RH settings.

**Results:**

105 residents who fell one or more times during 2009–2010 were identified with a mean age of 89 years, a mean of nine scheduled medications and seven diagnoses, and 83 % were women. Residents in LTC were ostensibly at higher risk for falls relative to those in RH settings as suggested by higher proportion of residents with multiple falls, multiple comorbidities, comorbidities that increase fall risk and visual impairment. Post fall injuries were sustained by 42 % of residents, and residents in RH sustained more injuries relative to LTC residents (47 vs 34 %). Use of FRIDs such as benzodiazepines, antipsychotic, antidepressant and various antihypertensive drugs was common in the present sample. No medication regimen changes were noted in the 6-month post fall period.

**Conclusions:**

The present study documented common use FRIDs by LTC and RH residents with multiple falls. These potentially modifiable falls risk factors are not being adequately addressed in contemporary practice, demonstrating that there is much room for improvement with regards to the safe and appropriate use of medications in LTC and RH residents.

## Background

Falls are a pervasive problem among older people, especially those living in long-term care (LTC) and assisted living [also known as retirement home (RH)] settings [1, 2]. One third of community dwelling older (>65 years) adults fall at least once per year, increasing to 50 % among those aged ≥80 years and over 60 % for those living in LTC settings [3–6]. Furthermore, long-term care dwellers have more than twice higher risk for falls, having a mean 1.7 falls per person-year compared to those living in community settings who have a mean of 0.65 falls per person-year [1]. Falls are a leading cause of significant morbidity and mortality in older people, lead to poor overall functioning, represent 85 % of injury-related hospital admissions in this age group and are the most common cause for emergency department visits by LTC residents [1, 2, 7]. Injurious falls may be devastating for those suffering outcomes such as hip fractures (falls account for 95 % of all hip fractures among older people in Canada), subsequent partial or permanent post-fall disability, or permanent institutionalization [2].

Falls in older people are due to multiple intrinsic (e.g., cognition, mobility) and/or extrinsic (e.g., environment, medications) risk factors, the latter of which are of particular interest, as medication related fall risk factors are well described in the literature [8–12]. Specifically, taking ≥4 medications is associated with an increased fall risk, as are certain medication types (e.g., psychotropic medications, blood pressure lowering medications) have been consistently associated with an increased fall risk [8–10, 13]. In addition, use of multiple medications in older people is commonplace with 2/3 of community dwelling older people taking 5 or more medications, while their long term care counterparts consume an average of 10 medications daily [14]. As such, it may be argued that medications are one of the most important risk factors for falls given their high rate of use and the potential for their modification [8, 9, 15]. Indeed, modifying high risk medications can lower fall risk and fall rates in older people. For example, in a randomised, controlled trial of psychotropic medication withdrawal and home based exercise program to prevent falls, Campbell et al. [16] observed a relative hazard for falls of 0.34 (95 % CI 0.16, 0.74) in the medication withdrawal group. Similarly, van der Velde et al. [17] observed a hazard ratio of 0.48 (95 % CI 0.23, 0.99) in a group of geriatric outpatients who had fall risk increasing drugs (FRIDs) withdrawn versus those whose medication regimens remained unchanged. Thus, there is support to the notion that modifying high-risk medication regimens can decrease fall risk and fall rates in older people [18]. In addition, failure to consider medications as possible causes/contributors to falls and failure to translate research evidence into practice can hamper any proposed comprehensive fall reduction strategies [9, 15].

Previous studies demonstrating associations between certain medications and their association with fall risk have documented baseline levels of psychotropic or cardiovascular medication use, yet there are relatively few contemporary studies that describe current trends of FRID use among older residents with a documented fall history in Canadian LTC facilities and/or in RH settings [19–21]. For example, Neutel et al. [21] documented the use of antidepressant (64 %), benzodiazepine (61 %), other sedatives (64 %), cardiovascular medications (58 %) and diuretics (59 %) in a sample (n = 125) of LTC residents who fell. It is logical to focus efforts in a high risk population such as older people in LTC settings, but it is also important to include people at risk in RH settings, as they are higher functioning and are more likely to be ambulating independently. As such, it is vital to avoid falls that may lead to fractures and functional impairment ultimately resulting in a need for permanent LTC placement. Furthermore, given the potential to reduce fall risk by modifying high-risk medications, it is important to assess whether or not a fall might trigger a proper and thorough medication review in these settings, as prior studies in other settings have documented continued use of FRIDs among older people who have fallen or sustained a hip fracture, which ostensibly represents a missed opportunity to improve the care of these vulnerable people [22, 23].

Existing data are also limited in that they do not provide detailed insights at the facility level or within the context of everyday practice. Appropriate management of residents who sustain a fall requires a diligent post fall assessment, including careful review of potentially modifiable risk factors, including medications. It is therefore essential to assess contemporary patterns of FRID use as well as current patterns of clinical care for residents who fall in order to identify opportunities to improve the care of older people [1, 11, 24]. There is also a need to understand current practices at the facility level to conceptualize rational solutions to improve any identified problems and identify desirable practices that should be continued. Previous studies have not documented these important issues. The present study sought to characterize medication related fall risk factors in LTC and RH residents who sustained one or more falls and to explore post fall medication use in these residents over a 1 year time period.

## Purpose

The objectives of this study were to (1) describe current patterns of FRID use in LTC and RH residents who sustained one or more falls during a 1-year time period (2009–2010) and (2) to explore current post-fall practices as they relate to the on-going use of FRIDs.

## Design and methods

### Setting, study participants, and information sources

This was a cross-sectional study of residents from a private, for-profit, retirement facility in Ontario, Canada with a continuum of care ranging from independent living to assisted care [referred to as retirement home (RH)] and long term care with a census of 311 beds (95 LTC and 216 RH beds). This facility is one of 13 retirement facilities in this region operated by a senior living organization providing services to approximately 3309 older residents in South-western Ontario. Each facility maintains an on-going registry of incident falls as part of routine clinical care protocols. Each fall case report form is completed by any nursing home staff who first becomes aware of the fall, then the form is immediately sent to the facility kinesiologist and charge nurse for post-fall assessments. This registry was used to identify study participants who were residents experiencing one or more incident falls between July 31, 2009 and July 31, 2010 from one facility. Incident cases of falls during the study period were included if the resident was 65 years of age or older. A fall was defined as any unexpected event in which the resident comes to rest on the ground, floor, or lower level [25].

A standardized case report form was developed for chart abstraction purposes. A research assistant collected data from three sources: (1) the electronic medical record, (2) medication administration records, and (3) the falls registry case report form. The following information was collected for each participant: age, vital signs, gender, living status (i.e., long term care or retirement home setting), list of medical conditions, list of current medications (scheduled and as needed), results of most recent laboratory test results, resident’s emotional state before the fall if observed, from fall report, perceived cause of the fall as per nursing note on fall report, any injury resulting from the fall, post-fall status, and, additional follow up required as a result of the fall. Any changes in medical status (e.g., recent infections or exacerbations of underlying diseases) or in medications that occurred in the 1-month period preceding the fall were also noted. Lastly, each resident’s chart was reviewed in detail during the immediate 6 month post-fall period to identify any aspects of care planning that included addressing medications as potential targets for intervention and/or referral to the consultant pharmacist for review to lower future fall risk. In addition, the medication dispensing records for these residents were reviewed during this 6-month period to ascertain any medications changes that might have occurred, but not otherwise documented in the progress notes. Fall risk increasing drugs were defined as medications that are known to be associated with an increased risk for falls such as antihypertensives, narcotic analgesics, and various psychotropic medications [8, 11–13]. The presence of any medication known to increase fall risk was noted from each resident’s medication dispensation data and cross-referenced with their medication administration record, and these data were captured in the case report form.

Data were entered into a Microsoft Access database designed for this project. Given the descriptive nature of this study, analyses were largely limited to descriptive statistics, though limited inferential testing was conducted (Chi Squared tests) using Epi-Info-7 software to compare FRID among residents by number of falls and place of residence. This study was approved by the University of Waterloo’s Office for Research Ethics, and individual consent from subjects was not required for this study.

## Results

We identified 105 residents who sustained one or more falls during the study period. These individuals had an average age of 89 (range 61–103) years, 83 % were female, with an average number of seven diagnoses and nine regularly scheduled medications (Table 1). The proportion of residents who fell more than once during the one-year study period was 46, and 27 % had a history of multiple falls in the 6 months preceding the first fall in the study period. There were more residents in the retirement home section (n = 64) versus long-term care (n = 41). Compared to retirement home dwelling residents, those living in long term care had a higher proportion of: residents with a history of one or more prior falls (61 vs 36 %), more than one fall during study period (60 vs 37 %), multiple comorbidities (8 vs 5), conditions associated with increased fall risk such as dementia (63 vs 34 %), osteoarthritis (44 vs 23 %), depression (42 vs 19 %), and visual impairment (63 vs 6 %). Residents in retirement home received an average of 9 regularly scheduled medications compared to 8 for LTC residents.

**Table 1:** Demographic characteristics of study sample

Characteristic	N (%)	Long term care (n = 41)	Retirement home (n = 64)
Gender			
Male	18 (17)	5 (12)	13 (20)
Female	87 (83)	36 (88)	51 (80)
Mean (SD) age (range)	89 ± 7 (61–103)	89 ± 8 (61–98)	89 ± 6 (67–103)
Use of cane or walker	71 (68)	24 (59)	47 (73)
Use of wheelchair	33 (31)	25 (6)	8 (13)
Visual impairment	30 (29)	26 (63)	4 (6)
Hearing impairment	21 (20)	20 (49)	1 (2)
Medications per resident (mean, SD, range)	9 (4), 1–22	8 (4), 1–18	9 (4), 1–22
Diagnosis per resident (mean, SD, range)	7 (3) (1–15)	8 (3) 3–15	5 (2), 1–9
Number who experienced 1 fall during study period	56 (54)	16 (40)	40 (64)
Number who experienced >1 fall during study period	47 (46)	24 (60)	23 (37)
History of falling in the last 6 months			
Has fallen 1–2 times	20 (19)	10 (24)	10 (16)
Multiple history of falling	28 (27)	15 (37)	13 (20)
No history	57 (54)	16 (41)	41 (65)
Diagnoses			
Hypertension	65 (62)	39 (95)	26 (40)
Osteoarthritis	33 (31)	18 (44)	15 (23)
Osteoporosis	20 (19)	10 (24)	10 (16)
Dementia	48 (4 6)	26 (63)	22 (34)
Depression	29 (28)	17 (42)	12 (19)
Stroke/TIA	28 (27)	15 (37)	13 (20)
Anemia	17 (16)	14 (34)	3 (5)
Cataracts	15 (14)	10 (24)	5 (8)
Anxiety	12 (11)	10 (24)	2 (3)
DM	12 (11)	6 (15)	6 (9)
Hypothyroidism	12 (11)	5 (12)	7 (11)
Atrial fibrillation	10 (10)	4 (10)	6 (9)
GERD	10 (10)	7 (17)	3 (5)
IHD	6 (6)	3 (7)	3 (5)
CPS score			
0–2	11 (28)	10 (26)	1 (100)
3–4	24 (62)	24 (63)	0 (0)
5–6	4 (10)	4 (11)	0 (0)

Numbers above may not add up to final sample due to missing data

CPS score 0–2 = intact cognition to mild cognitive impairment; CPS 3–4 = moderate to moderately severe cognitive impairment; CPS 5–6 = severe to very severe cognitive impairment; CPS scores available for 39 residents; CPS scale not routinely done in RH setting

**Fig. 1:**
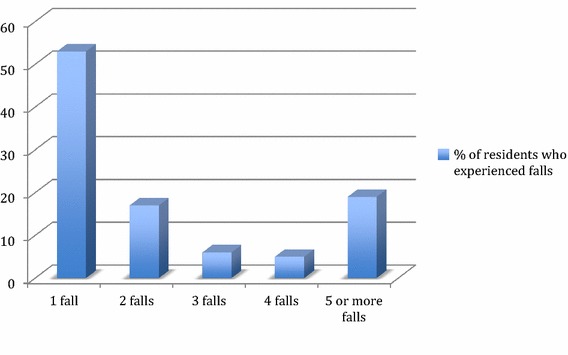
Frequency of falls among 105 residents

The number of residents experiencing one or more falls is summarised in Fig. 1. During the study period, 46 % of residents sustained more than one falls, with an overall average of 2.2 falls/resident and 3.7 falls/resident for those falling 2 or more times. The characteristics of the falls are summarised in Table 2. Overall, 42 % of residents sustained some type of injury, 24 % aches and pains, and 2 % sustained a fracture. The proportion of residents sustaining injuries was higher for RH (47 %) versus LTC setting (34 %).

**Table 2:** Characteristics of falls (N = 105 residents)

Characteristic	N (%)	Long term care (n = 41) n (%)	Retirement home (n = 64) n (%)
Injury or pain			
Aches/pain	24 (24)	6 (16)	18 (29)
Bruising/bumps	22 (22)	8 (21)	14 (23)
Skin tear/cut	20 (20)	6 (16)	14 (23)
Fracture/break	2 (2)	0 (0)	2 (3)
No injury	44 (44)	22 (58)	22 (36)
Location			
Activity room	1 (1)	0 (0)	1 (2)
Balcony	1 (1)	1 (3)	0 (0)
Bathroom	15 (16)	3 (8)	12 (20)
Bedroom	53 (55)	20 (54)	33 (55)
Dining room	4 (4)	2 (5)	2 (3)
Hallways	11 (11)	4 (11)	7 (12)
Lounge	7 (7)	6 (16)	1 (2)
Other	5 (5)	1 (3)	4 (7)
Prior activity			
Lying down	3 (3)	2 (6)	1 (2)
Eating	1 (1)	1 (3)	0 (0)
Reaching	7 (8)	2 (6)	5 (9)
Sitting	6 (7)	2 (6)	4 (7)
Sleeping	1 (1)	1 (3)	0 (0)
Standing	8 (9)	2 (6)	6 (10)
Tidying room	1 (1)	1 (3)	0 (0)
Transfer	11 (12)	2 (6)	9 (16)
Turning	5 (5)	1 (3)	4 (7)
Walking	37 (40)	15 (46)	22 (38)
Unknown	11 (12)	4 (12)	7 (12)
Emotional state			
Agitated	2 (3)	1 (4)	1 (2)
Alert	13 (18)	9 (32)	4 (9)
Anxious	5 (7)	3 (11)	2 (4)
Calm	28 (38)	17 (61)	11 (24)
Confused	9 (12)	6 (21)	3 (7)
Depressed	1 (1)	0 (0)	1 (2)
Sleepy	5 (7)	1 (4)	4 (9)
Unknown	31 (42)	4 (14)	27 (59)
Unwitnessed	1 (1)	0 (0.0)	1 (2)

Numbers above may not add up to final sample due to missing data

Most (65 %) falls occurred in bedrooms or bathrooms while 21 % occurred in common areas such as the dining room, hallways, or lounges. The most common activities prior to falling included walking (40 %), transferring (12 %), and turning or standing (12 %). There were some differences between RH and LTC residents as there were more of the former standing (10 vs 6 %) or transferring (16 vs 6 %) at the time of the fall. The emotional/cognitive state of the resident was noted in 74 cases and was most commonly documented as calm or alert (55 %), confused or sleepy (19 %), or agitated or anxious (9 %).

Use of fall risk increasing drugs was common in this sample. Statistically significant differences were noted for use of antidepressant (82 vs 57 %, *p* < 0.05) and antipsychotic drugs (39 vs 20 %, *p* < 0.05) for residents with one fall versus those who sustained >1 fall (Table 3), while statistically non-significant differences in the proportion of residents receiving diuretics (43 vs 29 %) and narcotics (21 vs 27 %) were also observed. When comparing RH residents to those living in LTC (Table 4), those living in RH used significantly less antipsychotics (15 vs 46 %, *p* < 0.05) while statistically non-significant differences were noted for SSRIs (34 vs 42 %) and trazodone (11 vs 20 %). Conversely, residents in LTC used significantly less narcotic medications (10 vs 33 %, *p* < 0.05) and less antihypertensive medications (*p* > 0.05) (Table 4). Lastly, 11 % of residents were receiving anticonvulsants, while use of tricyclic antidepressant and anticholinergic drugs was low (5 % each).

**Table 3:** Frequency of falls risk increasing medication use for single versus multiple falls

Medication/classification	Residents with 1 fall (n = 56 residents) N (%)	Residents with >1 fall (n = 49 residents) N (%)
Benzodiazepine receptor agonists	16 (28)	22 (44)
Antihypertensive drugs		
Beta-adrenergic antagonists	16 (29)	14 (30)
Calcium-channel antagonists	15 (27)	6 (13)
Diuretics	16 (29)	20 (43)
Renin–angiotensin–aldosterone system inhibitors	20 (36)	19 (40)
Antidepressants*		
Serotonin reuptake inhibitors	16 (29)	23 (47)
Trazodone	6 (11)	9 (19)
Tricyclic and other norepinephrine-reuptake inhibitors	3 (5)	2 (4)
Serotonin and norepinephrine-reuptake Inhibitors	2 (4)	2 (4)
Mirtazapine	5 (9)	4 (9)
Antipsychotics*		
Typical	0 (0)	2 (4)
Atypical	11 (20)	17 (35)
Narcotics	15 (27)	10 (21)
Digoxin	2 (4)	2 (4)
Anticholinergics	4 (7)	5 (10)

* *p* < 0.05 for comparison of residents with 1 fall versus >1 fall. Comparisons are for drugs as group, i.e., all antidepressants combined

**Table 4:** Frequency of falls risk increasing medication use according place of residence

Medication/classification	Long term care (n = 41 residents) N (%)	Retirement home (n = 64 residents) N (%)
Benzodiazepine receptor agonists	15 (36)	22 (34)
Antihypertensive drugs		
Beta-adrenergic antagonists	8 (20)	22 (34)
Calcium-channel antagonists	7 (17)	14 (22)
Diuretics	10 (24)	26 (41)
Renin–angiotensin–aldosterone system inhibitors	16 (39)	23 (36)
Antidepressants		
Serotonin reuptake inhibitors	17 (42)	22 (34)
Trazodone	8 (20)	7 (11)
Tricyclic and other norepinephrine-reuptake Inhibitors	1 (2)	4 (6)
Serotonin and norepinephrine-reuptake Inhibitors	2 (5)	2 (3)
Mirtazapine	3 (7)	6 (9)
Antipsychotics*		
Typical	0 (0)	2 (3)
Atypical	19 (46)	8 (13)
Narcotics*	4 (10)	21 (33)
Digoxin	1 (2)	3 (5)
Anticholinergic drugs	6 (14)	3 (5)

* *p* < 0.05 for comparison of residents residing in long term care versus retirement home. Comparisons are for drugs as group, i.e., all antidpressants combined

Of note, vitamin D and calcium were used by 43 and 34 % of residents, respectively, while bisphosphonates were used by 66 % of residents with osteoporosis. A thorough review of resident’s charts did not reveal any evidence that medication related causes were investigated regardless of how many falls the resident sustained. Furthermore, additional review of medication administration records did not reveal any medication regimen changes in the 6-month period after each fall that was related to medications that might have played a role in falls. No pharmacy consultant notes could be located for any of these residents during the same time period.

## Discussion

We found that LTC and RH residents with a recent fall were commonly receiving multiple FRIDs and multiple medications. Patterns of FRID use differed somewhat between those who sustained 1 versus >1 fall, and between residents living in LTC versus RH settings. Overall, residents who sustained >1 fall had a high burden of FRIDs relative to residents with only one fall as demonstrated by higher proportions of benzodiazepine, diuretic, renin-angiotensin-aldosterone inhibitors, antidepressant, and antipsychotic drugs. Our sample had a predominance of women, a significant burden of fall risk factors such as polypharmacy, previous falls, dementia, older age, sensory impairments, use of assistive ambulatory devices, and a high prevalence of post fall injuries.

Benzodiazepine, antidepressant, and antipsychotic medication use among LTC residents was common, consistent with prior findings in LTC settings [20, 21, 26]. Conversely, antihypertensive drugs, narcotics, vitamin D and/or calcium supplements were less commonly used in LTC residents. The latter two observations are consistent with studies documenting low rates of analgesic and anti-osteoporotic drug use in this setting [27, 28]. Vitamin D has been associated with a reduced risk for falls, is well tolerated and inexpensive, and thus represents an opportunity to reduce fall risk in this vulnerable population [2, 29]. Similarly, use of supplemental calcium could also be improved given the high prevalence of osteoporosis in this setting [30]. The lower rates of antihypertensive medication and narcotic use in LTC residents compared with RH residents may represent a conscious effort on the part of prescribers to avoid using these medications in an ostensibly frail population. Alternatively, lower rates of narcotic use in LTC residents may be due to inadequate recognition of pain, especially among residents with cognitive impairment [31].

With regards to the role that consultant pharmacists could have played in falls risk reduction strategies, our findings were revealing. Review of the resident’s charts revealed no medication regimen reviews from consultant pharmacists that addressed fall risk reduction, nor were such reviews requested by staff post-fall to assess the potential contribution of medications to falls. During discussions with the principal investigator, consultant pharmacists noted that every quarter, they received a list of residents who had fallen, but they felt that this time lag made subsequent medication regimen reviews futile. The pharmacists also noted that their consult notes are often misplaced (it is unclear why this is so) and thus seldom reach the attending physician. It was also apparent that medication regimen reviews were not being conducted in a systematic manner that could otherwise aid in identifying potentially modifiable medication related fall risk factors [9, 15, 32]. Lastly, pharmacists noted that falls care plans for residents who had fallen did not include pharmacy, and that pharmacists were not part of these care conferences. As such, pharmacists appeared to be working in isolation rather than as part of the interdisciplinary team.

These findings are concerning, as in Ontario the Ministry of Health requires and reimburses pharmacists to conduct on-going medication reviews for nursing home residents and to participate in interdisciplinary meetings to evaluate medication regimens. The quarterly medication regimen review would appear to present an ideal opportunity to address medication related issues of relevance to falls, as medications represent one of the most modifiable risk factors for falls. Indeed, prior studies in community settings have demonstrated that reducing FRIDs can result in a 39–66 % reduction in falls which is of relevance to RH residents in the present study [16, 17, 33–35].

Indeed, a fall in either the NH or RH setting should trigger a thorough and systematic medication regimen review as part of a comprehensive care plan, with particular attention to blood pressure lowering drugs, psychotropic drugs, and optimization of vitamin D and calcium status, though it is clear that further research is necessary to clarify the role of medication based programs as a tool for reducing fall risk, and ultimately, falls [9, 15, 29].

On a positive note, consultant pharmacists suggested their awareness of potentially modifiable medication related risk factors could be improved if a simple algorithm existed that could assist them during their medication regimen reviews, and that they would use such a tool in their practice. This suggestion proved fruitful, and given the absence of a brief and practical tool, our group developed and pilot tested a brief algorithm to reduce fall risk attributable to FRIDs [15]. Use of this this instrument was found to be feasible in practice, and led to an absolute increase of 20 % in pharmacist’s suggestions related to fall risk reduction. It was also pointed out that use of supplemental calcium decreased dramatically in LTC facilities following publication of studies demonstrating an association between calcium supplement use and increase risk of vascular events, despite major limitations of these data [36]. Such information has proved valuable, as it has identified areas for future education and intervention efforts aimed at lowering fall risk and optimising the use of consultant pharmacy services.

## Limitations

There are inherent limitations in retrospective studies. The reasons behind the lack of thorough medication regimen reviews post fall, especially in residents with more than one fall could not be ascertained from the medical records. Sample size may be considered as a limitation in terms of generalizability and statistical ability to detect differences between groups, yet our study provides more granular data compared with database studies, in particular with regards to proximal outcomes of falls, prior activity, and falls in a RH setting, where data thus far are scant. In addition, these data might be ostensibly generalizable to the other 12 facilities (total census 3309) as they are all operated by the same organisation with identical standard operating procedures. The exact role that FRIDs played in these resident’s falls could not be ascertained from this study. It should be stressed that this is no different from clinical practice. In practice, clinicians are left to sort through potential contributors to falls after a person has fallen, in much the same manner as in the present study. We relied on a single source of falls data to identify residents, but have no data describing the accuracy of the falls incident reporting system used in this facility, thus it is possible that falls are underreported. Lastly, we were unable to ascertain dietary intake of calcium and vitamin D.

This study nonetheless offers a contemporary and detailed analysis of use of FRIDs in a vulnerable sample of LTC and RH residents who have fallen. These data represent an important step in understanding where to focus future quality improvement efforts aimed at reducing modifiable falls risk factors, with the ultimate goal of reducing falls across the 12 facilities of the broader organization, which provides services to approximately 3080 older residents in south-western Ontario, Canada.

## Implications for practice and research

There are many implications for practice, and perhaps the most salient is the necessity for consultant pharmacists to adopt a systematic approach to resident care, which would be helpful in identifying medication-related fall risk factors [10, 15, 32]. Consistent with known associations between various medications and falls, psychotropic drugs, antihypertensives, and the use of multiple medications should remain in the forefront of fall risk reducing strategies for LTC residents who experience falls. Similarly, for residents with multiple falls, psychotropic drugs and blood pressure lowering drugs are likewise good targets for fall risk reduction [16, 17, 37–39]. Lastly, optimising calcium and vitamin D intake (through any means possible) represent a parsimonious opportunity to reduce fall risk and improve bone health. In addition, while it may be useful for falls incident reports to be shared with pharmacists in a more timely manner (e.g., within one week of the fall), potentially modifiable risk factors can still be identified and addressed even if medication regimen reviews are conducted at a later date, especially in light of our findings that no reviews appeared to be conducted even among residents who fell multiple times.

From the research perspective, additional work will be conducted to further develop and test our falls algorithm instrument on a larger scale, establish its utility in practice, and study the ability of this instrument to help pharmacists reduce the incidence of falls. In addition, while it is difficult to assess the potential contribution of medications to falls in individual patients in practice, there is a need to develop methods that may assist pharmacists in identifying the most likely drug(s) that may be contributing to falls and which medications might be superfluous, which in turn would aid in the overall care plan for these residents. Lastly, despite the success of multifactorial interventions in reducing falls in LTC such interventions are not routinely or readily incorporated into practice [8–10, 40]. It is possible that these programmes are too time or labour intensive, or that the closed system LTC infrastructure does not readily permit uptake of these models. Indeed, Haralambous et al. [24] recently suggested that an action research approach is useful in the context of engaging staff in falls prevention strategies that are sustainable and contextually appropriate. In this regard, long-term care facilities represent ideal settings to continue to investigate novel methods to lower fall risk and falls. Given the challenges associated with complex interventions that may not be readily sustainable, it may be possible to improve fall risk reduction by using multidisciplinary health care team members who are already providing care to these residents, namely the consultant pharmacist. Lastly, results from this research project are being utilized as part of a multifaceted clinical research program to lower fall risk and ultimately lower fall rates by optimising medication use in this vulnerable population. This program also involves multiple health care professionals and addresses systems issues aimed at developing practice based changes that are sustainable over the long term.

## Conclusion

In conclusion, the present study documented common use of FRIDs by LTC and RH residents with multiple falls. These potentially modifiable falls risk factors are not being adequately addressed in contemporary practice, demonstrating that there is much room for improvement with regards to the safe and appropriate use of medications in LTC and RH residents and lowering resident fall risk.

## Abbreviations

LTC: long term care
RH: retirement home
FRIDs: fall risk increasing drugs
